# VE1 Immunohistochemistry Improves the Limit of Genotyping for Detecting *BRAF*^V600E^ Mutation in Papillary Thyroid Cancer

**DOI:** 10.3390/cancers12030596

**Published:** 2020-03-05

**Authors:** Sonam Choden, Somboon Keelawat, Chan Kwon Jung, Andrey Bychkov

**Affiliations:** 1 Department of Pathology, Faculty of Medicine, Chulalongkorn University, Bangkok 10330, Thailand; choden.sonam90@yahoo.com (S.C.); trcskl@gmail.com (S.K.); bychkov.andrey@kameda.jp (A.B.); 2 Department of Hospital Pathology, College of Medicine, The Catholic University of Korea, Seoul 06591, Korea; 3 Cancer Research Institute, College of Medicine, The Catholic University of Korea, Seoul 06591, Korea; 4 Department of Pathology, Kameda Medical Center, Kamogawa, Chiba 296-8602, Japan; 5 Department of Pathology, Nagasaki University Graduate School of Biomedical Sciences, Nagasaki 852-8523, Japan

**Keywords:** BRAF, immunohistochemistry, papillary thyroid carcinoma, VE1, diagnostic performance

## Abstract

Detection of *BRAF*^V600E^ is useful for making diagnosis and risk stratification of papillary thyroid carcinoma (PTC). Molecular testing, however, is not always available for routine clinical use. To assess the clinical utility and reliability of VE1 immunohistochemistry (IHC) for detecting *BRAF*^V600E^ mutation in PTC, VE1 IHC was performed on the tissue microarrays of 514 patients with PTC and was compared with Sanger sequencing results. Of 514 PTC cases, 433 (84.2%) were positive for VE1 expression. Among 6 discordant cases between VE1 IHC and Sanger sequencing, 3 initial VE1-false negative cases turned out to be true false negative on repeat testing, and 3 VE1-false positive cases showed *BRAF*^V600E^ mutation using digital PCR analysis. PTCs with low variant allele fraction were positive for VE1 IHC but were not detected using sequencing. VE1 IHC showed 99.3% sensitivity, 100% specificity, 100% positive predictive value, and 96.4% negative predictive value. The *BRAF*^V600E^ mutation was significantly associated with older age, multifocality, extrathyroidal extension, lymph node metastasis, and advanced tumor stage. In conclusion, VE1 IHC is a reliable method for detecting *BRAF*^V600E^ mutation in PTC specimens.

## 1. Introduction

Thyroid cancer is the most common endocrine tumor accounting for 3% of all new malignancies worldwide [1]. Currently thyroid carcinoma occupies the fifth rank by incidence among cancers in women [1]. The incidence of thyroid cancer has been increasing rapidly over the past few decades due to the widespread use of high-resolution imaging facilities which are able to detect small-sized low-risk cancers [2]. In the near future, thyroid cancer is estimated to enter the top three of the most common malignancies in females [3]. Fortunately, papillary thyroid carcinoma (PTC) is known to have an excellent prognosis with a 10-year survival rate of >95% [4]. PTC is the major histological subtype, which accounts for 85–90% of all thyroid cancers [5,6].

B-type Raf Kinase (*BRAF*) mutations are well-known oncogenic drivers associated with various solid tumors including thyroid carcinoma, colorectal carcinoma, melanoma, ovarian cancer, and others [7]. Although more than fifty *BRAF* mutations have been identified, *BRAF*^V600E^ point mutation accounts for more than 90% of those mutations [8]. *BRAF*^V600E^ mutation is the most common genetic alteration in PTC [5]. *BRAF*^V600E^ mutation is associated with aggressive clinicopathological characteristics of PTC such as lymph node metastasis, distant metastasis, higher tumor staging, extrathyroidal extension, loss of radioiodine avidity, and higher tumor recurrence and, therefore, represents an adverse prognostic factor in PTC [9,10,11]. *BRAF* mutation status in PTC might be helpful to stratify the risk of patients more accurately and to adjust management algorithms—from preoperative planning to postoperative decisions regarding radioiodine therapy appropriate surveillance modalities [8,12]. *BRAF*^V600E^ mutation status is also important in developing therapeutic targeting and predicting patient outcome in response to targeted therapy [13,14].

The prevalence of *BRAF*^V600*E*^ mutation in PTC largely depends on the target population and certain clinicopathological variables, such as age, histological type, and others. *BRAF*^V600*E*^ mutation is found in almost half (45–50%) of PTC cases in the Western series originated from the USA and Europe [5,10]. However, in the Asian population, prevalence is more heterogeneous with a wide range of reported rates spanning from 31% to 87% [15,16].

There are different DNA-based techniques available for the detection of *BRAF*^V600E^ mutation in PTC [17]. Sanger sequencing has been widely utilized as the gold standard method; however, it has a reported detection sensitivity of only 10–20% of mutant allele frequency, and therefore, this method alone might not be sufficient to detect the mutation in cases with low tumor cellularity [18,19,20]. Pyrosequencing and real-time polymerase chain reaction (PCR) are known to be more sensitive than Sanger sequencing, with a reported detection sensitivity of 5% and 1% mutant alleles, respectively [21,22,23]. Since different techniques have different performance rates, the methods employed to detect the mutation might have a significant impact on the prevalence rate of the mutation.

Although molecular testing, particularly Sanger sequencing, has been widely acknowledged as the gold standard for the detection of *BRAF*^V600E^ mutation, molecular workup remains an expensive, labor- and time-intensive process, requiring a molecular laboratory to be established, which is not always feasible in resource-limited settings [24,25]. Immunohistochemistry (IHC) with mutation-specific antibodies, sometimes acknowledged as a new generation IHC, has had increased practical utility in the last 5–10 years [26]. Some examples of the mutation-specific antibodies are IDH1 R132H for low-grade gliomas, EGFR and ALK for lung adenocarcinoma, SP174 for *RAS*-mutant tumors, and others [27,28,29]. Recently, a new approach for the detection of *BRAF* mutation by means of IHC has been established [30]. The anti-BRAF V600E (VE1) mouse monoclonal antibody was generated using the synthetic peptide designed against an 11-amino acid peptide representing the *BRAF*^V600E^ mutated amino acid sequence from amino acids 596 to 606 (GLATEKSRWSG) [30].

IHC equipment is widely available in the pathology laboratories and gives a faster result compared to molecular biology techniques, thus reducing the turnaround time between the physician’s request and the result. Several studies have been done on the performance of VE1 IHC to detect *BRAF*^V600E^ mutation, and most of the reports have confirmed excellent concordance between VE1 IHC and molecular genotyping, and suggested that VE1 IHC can be used as an alternative to *BRAF*^V600E^ genotyping [17,22,24,25,30,31,32,33,34,35]. Such results are applicable not only to PTC but also to other tumors harboring this mutation such as colorectal cancer, malignant melanoma, and brain tumors [36]. IHC detection of *BRAF* is the best-tailored method for a surgical pathology laboratory.

The main aim of this study was to assess the concordance rates between VE1 IHC and Sanger sequencing for detecting *BRAF*^V600E^ mutation. We also aimed to render discordant cases with additional molecular workup and to analyze clinical significance of *BRAF*^V600E^ in the Korean population known to be highly saturated with this mutation.

## 2. Results

### 2.1. VE1 Immunohistochemical Staining vs. Sanger Sequencing

**Figure 1:**
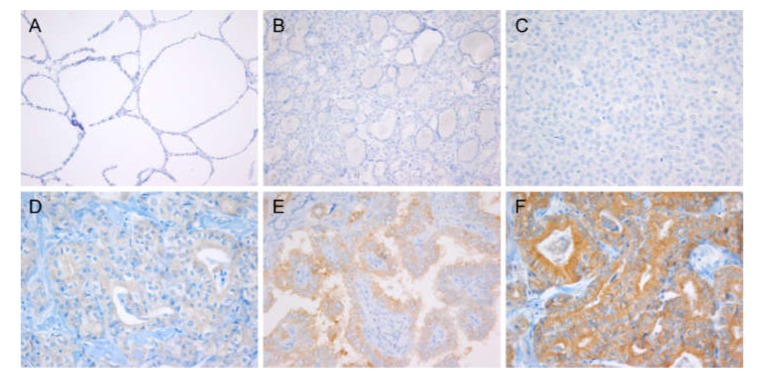
Representative images of BRAF V600E (VE1) immunostaining. Negative staining in normal thyroid tissue (**A**, ×200) and *NRAS*-mutant thyroid tumors (**B**, noninvasive follicular thyroid neoplasm with papillary-like nuclear features, ×200; **C**, follicular thyroid carcinoma, ×200). Papillary thyroid carcinoma (PTC) with *BRAF*^V600E^ showed variable staining ranged as weak (**D**), moderate (**E**), and strong (**F**) intensity detected in the cytoplasm of tumor cells; ×400.

Normal follicular cells adjacent to tumors (n = 24) did not express VE1 (Figure 1A). All non-PTC tumors (n = 71) with *RAS* mutation recruited as independent cohort for antibody validation were negative for VE1 IHC (Figure 1B,C). VE1 immunostaining of different intensity, i.e., weak, moderate, and strong was seen in cytoplasm of the PTC cells (Figure 1D–F). Most of the positive cases showed homogenous cytoplasmic staining, but some cases demonstrated heterogeneous staining with variable intensities and proportions.

**Figure 2:**
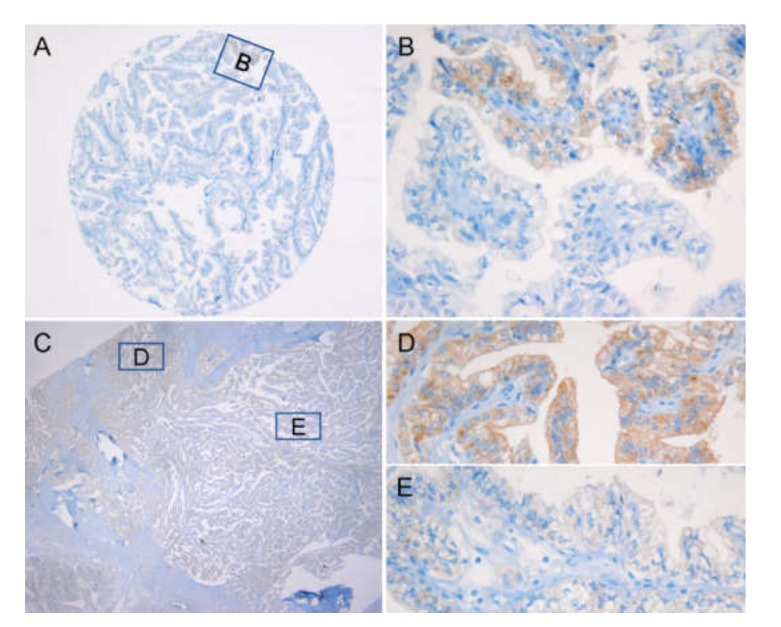
Heterogeneity of VE1 immunostaining. In the tissue microarray core, only a small cluster of cancer cells showed VE1 reactivity (**A**), which could be easily missed during sampling. The high-power view of the boxed area in Figure 2A shows a focal positive immunostaining (**B**). This variation from negative to strongly positive staining was further reproduced on the whole-tissue section (**C**–**E**); however, a proportion of VE1-positive cells was much higher; ×40 (A), ×100 (C), ×400 (B, D, E).

**Figure 3:**
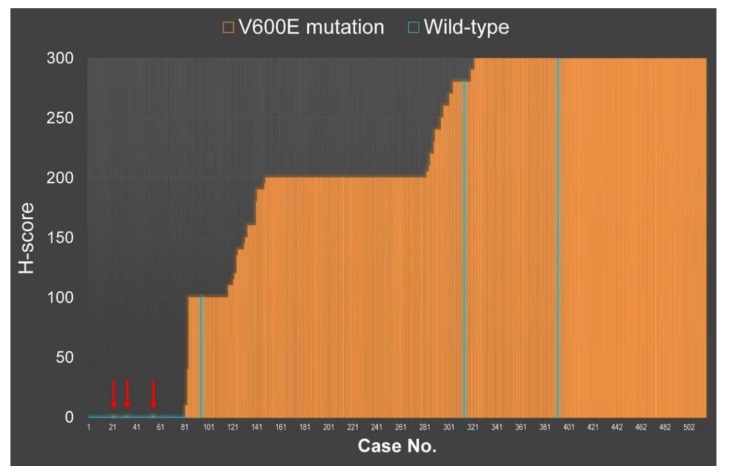
H-score for VE1 staining plotted against *BRAF* exon 15 genotype using Sanger sequencing. Arrows indicate 3 cases with initially VE1 false negative results. Three cases with initially VE1 false positive results (light blue lines on orange background) showed *BRAF*^V600E^ mutation using digital PCR.

Heterogeneity was best seen on the whole tissue sections (Figure 2). In the positive cases, H-scores ranged from 10 to 300 with mean and median H-scores of 238 and 260, respectively (Figure 3). Out of 514 PTC cases, 433 (84.2%) were positive for VE1 expression. Sanger sequencing of *BRAF* exon 15 detected *BRAF*^V600E^ mutation in 433 PTCs (84.2%) and *BRAF*^K601E^ in 1 (0.2%) case, whereas 80 (15.6%) cases were of wild type. A single case with *BRAF*^K601E^ mutation was negative for VE1 IHC. Despite immunostaining and direct sequencing revealed *BRAF* mutation in the same amount of cases (n = 433), six of them showed discordant results between both methods.

### 2.2. Evaluation of Discordant Cases

Of 6 initially discordant cases (Table 1, Figure 3), 3 cases were negative for *BRAF*^V600E^ using immunohistochemistry but positive using Sanger sequencing (false negative result), and 3 cases were positive for *BRAF*^V600E^ using VE1 immunostaining but negative using Sanger sequencing (false positive result). In cases where IHC and DNA sequencing were discordant, both tests were repeated on the whole tissue sections, and droplet digital PCR was performed for the validation of initially false positive cases.

**Figure 4:**
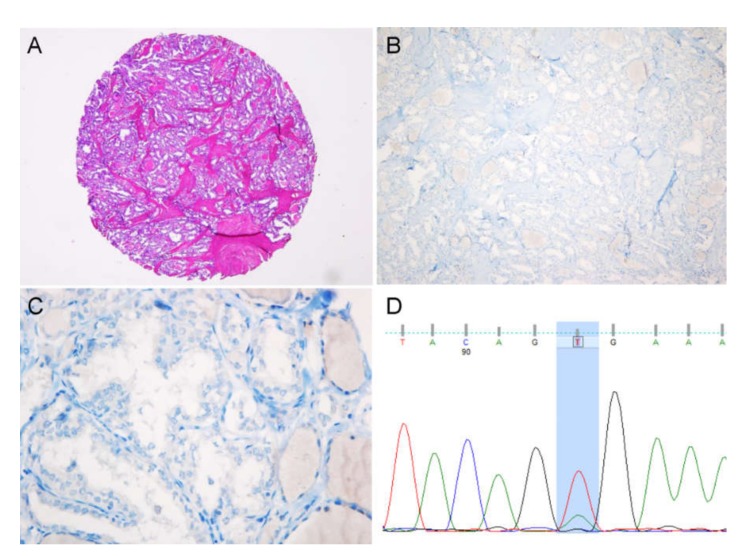
False negative VE1 immunostaining. A predominantly follicular-patterned PTC (**A**) was negative for VE1 immunostaining (**B, C**), whereas Sanger sequencing revealed heterozygous *BRAF*^V600E^ mutation (*BRAF* c.T1799A) (**D**); ×40 (A), ×100 (B), ×400 (C).

**Figure 5:**
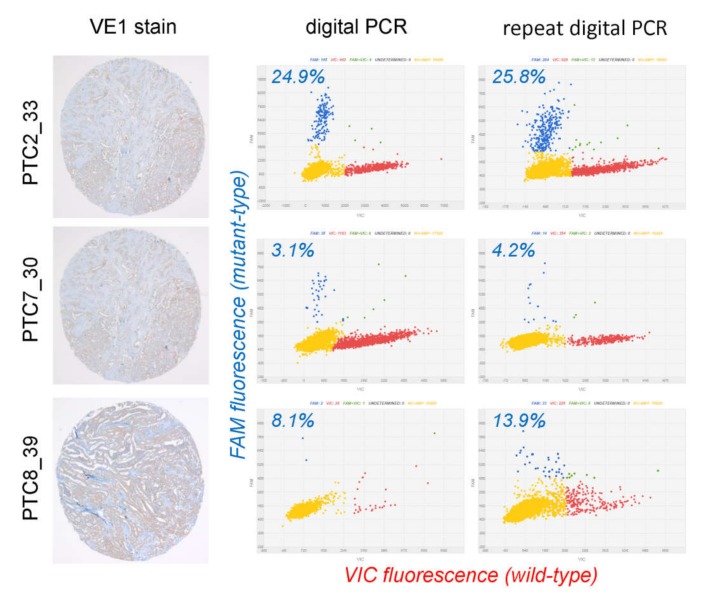
Droplet digital PCR analysis in discordant cases showing VE1 expression but wild type of *BRAF* using Sanger sequencing (false negative Sanger sequencing). All three cases turned out to be mutant type using digital PCR. Blue, red, green, and yellow dots represent the *BRAF*^V600E^ mutant allele, wild-type allele, both alleles, and none (no template), respectively. Variant allele frequency is shown as percentage. FAM, 6-carboxyfluorescein; VIC, 2’-chloro-7’phenyl-1,4-dichloro-6-carboxyfluorescein.

Of 3 cases with initially false negative results, all were repeatedly negative for VE1 immunostaining but positive for *BRAF*^V600E^ using Sanger sequencing (true false negative) (Figure 4). Of 3 cases with initially VE1 false positive result, all were successfully analyzed using digital PCR, which detected the presence of *BRAF*^V600E^ mutation (Figure 5). One case showed low level mutation (<5%). In the other two cases, although the mutant allele frequency was relatively high (8–26%), the values of variant allele frequency were not reliable because mutant and wild-type dots existed in the in-between and were not clearly separated from each other (Figure 5). The intermediate droplets (so-called “rain”) are mainly caused by fragmented and damaged DNA isolated from the formalin fixed tumor tissue (Figure 5). In summary, out of 6 discordant cases, 3 cases were resolved by repeat testing using a more sensitive method on whole tissue sections. Finally, after additional validation, VE1 immunostaining produced 3 false negatives but no false positives, as summarized in Table 1. 

### 2.3. VE1 Performance in PTC Tissue Microarray (TMA)

By considering Sanger sequencing (or digital PCR in the initially discordant cases, see above) as the gold standard method for detection of *BRAF*^V600E^ mutation, we calculated the analytical performance of VE1 immunostaining. VE1 IHC showed sensitivity and specificity of 99.3% (95% confidence interval [CI], 98.0% to 99.9%) and 100% (95% CI, 95.4% to 100%), respectively. The positive and negative predictive values were 100% and 96.4% (95% CI, 89.4% to 98.8%), respectively.

### 2.4. Clinical Correlation

Out of 514 PTCs, 436 had *BRAF*^V600E^ after reexamining the *BRAF* molecular testing. *BRAF*^V600E^ mutation using molecular testing was significantly associated with older age (*p* < 0.01), multifocality (*p* < 0.01), extrathyroidal extension (*p* < 0.01), lymph node metastasis (*p* = 0.01), higher pT category (*p* < 0.01), and advanced tumor stage (*p* = 0.01), as shown in Table S1. When VE1 IHC was considered as a reference test for *BRAF*^V600E^ status, all the variables above remained significant in the univariate model. Interestingly, we found no statistically significant difference between cases with high and low H-score (above vs. below median) with regard to all clinicopathological variables. The clinicopathological features of the 3 false negative cases using VE1 IHC are shown in Table S2.

## 3. Discussion

In this study, we compared the performance of VE1 IHC to detect *BRAF*^V600E^ mutation with Sanger sequencing. We assembled a TMA collection with a large series of PTC and found that 433/514 cases (84.2%) were positive using VE1 IHC. The same number of PTCs was *BRAF*^V600E^ mutant on direct sequencing; however, 6 cases showed discordant results between both methods. Additional molecular testing with droplet digital PCR was able to resolve 3/6 discordant cases. As a result, VE1 IHC showed excellent analytical performance in our series with 99.3% sensitivity and 100% specificity. Herein, we report the lessons learned from discordant cases in our set and available literature, and also the association of *BRAF*^V600E^ mutation with clinicopathological variables.

*BRAF* gene sequencing is the gold standard for *BRAF*^V600E^ detection, but it is a relatively expensive, multistep process, requiring sufficient quality DNA, and rather sophisticated setup. In contrast, IHC, which is widely used in diagnostic laboratories, is a simple and cost-effective method able to provide rapid results. Previous reports showed excellent concordance between IHC and molecular genotyping, and majority of the authors, including those of meta-analysis studies, suggested VE1 IHC as an alternative to *BRAF* sequencing [34,37].

Various DNA-based methods (Sanger sequencing, pyrosequencing, real-time PCR, SNaPshot, and others) have been employed in previous studies to correlate with results of VE1 immunostaining, with a direct sequencing being the most common [34,37]. A recent meta-analysis encompassing 29 studies found that IHC for BRAF VE1 is highly sensitive and reasonably specific in detecting the *BRAF*^V600E^ mutation in thyroid histopathology, which was illustrated by the pooled sensitivity and specificity of 96.8% and 86.3%, respectively [37]. This means, on the other hand, that about 5% of cases (1.2% in our study) were discordant. Interestingly, only a limited amount of previous studies addressed discordant cases either by repeat testing (both genotyping and IHC) or by employing more sensitive technique.

Although Sanger sequencing has been widely acknowledged as the standard technique for detection of point mutation, from the literature review, we found out that the discordant rates are much higher when using Sanger sequencing (7–23%) to validate the performance of VE1 IHC [20,23,25,31,35,38,39,40], than using a more sensitive molecular method, such as real-time PCR (0.1–8%) [19,22,23,41,42,43,44,45]. Several groups achieved a discordance rate of < 2% when VE1 immunostaining was compared to real-time PCR [22,43]. Jung et al. used RNA-ISH, and their discordant rate was 1.9% [41]. Sanger sequencing is regarded as the gold standard method for identifying oncogene in solid tumors, but it produced relatively higher discordant rates with higher false negative rates compared to IHC. Since different techniques inherently have different performance rates, the use of a single type of molecular method may increase the risk of false positive or false negative results [46]. Therefore, a combination of molecular studies may provide relatively less false negative and false positive results than using only a single method. For our study, we adopted Sanger sequencing and a digital droplet PCR method to validate VE1 IHC.

From our literature review, there were nine other studies that carried out re-genotyping to resolve discordant results from the initial validation, summarized in Table 2 [17,19,23,31,33,35,38,44,47].

Less than half (41%) of the discordant cases were fixed on the second round of genotyping or using an alternative technique. Bullock et al. initially had 11 discordant cases using Sanger sequencing alone, which dropped to 3 after re-Sanger sequencing and mass parallel sequencing [31]. McKelvie et al. reduced their discordant cases from 9 on using competitive PCR to 1 on using SNaPshot [47]. In our series, we could reduce discordant cases from 6 to 3 by using digital PCR as the second molecular method. Despite the successful examples above, 59% (45/76) of initially discordant cases remained unresolved after re-genotyping (Table 2). From the comparative analysis of IHC with three DNA-bases assays, failure to achieve a definite result was more frequently seen with Sanger sequencing than with other methods (pyrosequencing and SNaPshot).

Further analysis of the published studies showed that after matching with initial genotyping, VE1 false positives were 4.3 times more frequent than false negatives. However, almost half (29/61, 48%) of VE1 false positive cases were fixed after repeat molecular testing. In our series, all 3 VE1 false positive PTCs were successfully resolved using digital PCR, which detected the presence of *BRAF*^V600E^ mutation. On the other hand, VE1 false negatives could rarely be fixed by repeat testing, e.g., 0/3 in our study and 2/11 (18%) in the literature.

From the literature review, possible explanations for VE1 IHC false negatives could be technical (suboptimal fixation condition, tissue ischemia in perinecrotic zones) [38,48] or functional (loss of expression of mutation antigen, presence of additional mutation preventing translocation of mutated mRNA into functional protein) [43]. As it could be learned from our series, tumors with VE1 immunoreactivity but negative results of Sanger sequencing represented false negative sequencing but not false positive IHC. This particularly should be kept in mind when one encounters PTC cases with uniform and unequivocal staining with VE1 antibody. Such discrepancy usually arises due to an excess of wild type allele compared to the mutant allele when only the minority of cells in the sample submitted for sequencing are from tumor areas [38]. More sensitive or allele-specific methods are able to resolve this discordance (Figure 5). In addition, VE1 IHC false positives may be due to sample contamination, antigen cross-reactivity, or usage of an inappropriately high concentration of antibody [23,43].

Our study showed that VE1 IHC was able to detect the mutation in the formalin-fixed paraffin-embedded (FFPE) specimens with low tumor cellularity, low mutant allele frequency, and high tumor heterogeneity. Moreover, in our experience, prior decalcification doesn’t interfere with the IHC test results. Unfortunately, such decalcified specimens are not appropriate for molecular testing. Another important benefit of VE1 immunostaining is that it can be applied to small-sized tissue samples. There are several reports available on VE1 immunostaining in thyroid core needle biopsies and fine-needle aspirates, which was successfully used to detect *BRAF*^V600E^ before surgery [18,49,50].

We found that *BRAF*^V600E^ mutation was associated with older age, multifocality, tall cell morphology, extrathyroidal extension, lymph node metastasis, higher pT category, and advanced tumor stage. All the above clinicopathological parameters are known as adverse prognostic factors in thyroid cancer [40,44,51], which could imply that even in a population with a high *BRAF* prevalence in PTC, this mutation may serve as an adverse prognostic parameter. However, further analysis found no significant differences in recurrence and disease specific survival between patients with and those without *BRAF*^V600E^ mutation. Therefore, we would not claim prognostic significance of *BRAF*^V600E^ mutation based on this series. It should be noted that no microcarcinomas less than 1.0 cm were included in this study, which could be another reason to bias clinical correlation analysis.

## 4. Materials and Methods

### 4.1. Case Selection

We retrospectively reviewed the clinicopathological data of the patients who underwent thyroidectomy at Seoul St. Mary’s Hospital, the Catholic University of Korea. Surgically operated and morphologically verified PTC of all histological variants from 2008 to 2010 were included. We excluded cases with missing clinical and follow up data, tumors equal to or less than 10 mm, and low-quality samples not suitable for molecular and IHC analysis, such as those with extensive fibrosis, calcification, and hemorrhage. A total of 514 consecutive patients with PTC were enrolled in this study and their tissue blocks were selected for DNA extraction and VE1 immunohistochemistry. All pathology slides were reviewed by an experienced endocrine pathologist (C.K.J.) and classified following the diagnostic criteria and terminology of the World Health Organization [52]. Cancer staging was done using the American Joint Committee on Cancer (AJCC) staging system, 7th Edition [49]. This study was approved by the institutional review board of Seoul St. Mary’s Hospital (KC16SISI0104 and KC16SISI0709) and Faculty of Medicine, Chulalongkorn University Institutional Review Board (050/61). Informed consent was obtained from each patient.

### 4.2. Tissue Microarray Construction

Manual tissue microarrayer (QuickRay, Unitma Co. Ltd., Seoul, Korea) was used for TMA construction. All hematoxylin and eosin stained slides of PTC were reviewed by an expert endocrine pathologist (Chan Kwon Jung.) and a single slide with a representative tumor from each case was selected for TMA construction. The area corresponding to the selected area on the FFPE block was marked with a felt marker. One core from each case was punched out with a 2 mm diameter needle and arrayed into a recipient block. A distance of 1 mm was kept between cores.

### 4.3. VE1 Immunohistochemistry

Immunohistochemistry for VE1 was performed on 4 µm-thick TMA tissue sections using an automated Ventana BenchMark Ultra immunostainer (Ventana Medical Systems, Tucson, AZ, USA). Tissues sections were incubated with the anti-BRAFV600E (VE1) mouse monoclonal antibody (Ventana Medical Systems, catalog number 790-4855) for 16 min at 37 °C. Immunoreactivity of VE1 was visualized using an OptiView DAB IHC detection kit (Ventana Medical Systems) and then counterstained with Hematoxylin II and Bluing Reagent for 4 min. Human tonsil and placenta tissues were used as negative control tissue in parallel for each staining run (3 cores per each TMA block). Negative staining control was performed by replacing the primary antibody with normal serum.

To validate the specificity of the VE1 antibody, we performed the VE1 IHC in 71 *RAS*-mutant non-PTC tumors including follicular neoplasms, Hürthle cell neoplasms, and poorly differentiated carcinomas that were employed in our previous study [53].

The immunohistochemical scoring was done independently by two pathologists (Sonam Choden and Chan Kwon Jung) using the H-scoring system, blinded to the results of genotyping. H-score is a semi-quantitative system which includes both the proportion (0–100%, in 5% increments) and intensity of positive cells (0, absent; 1+, weak; 2+, moderate; 3+, strong staining). H-score range was obtained by combining intensity and proportion scores, as previously described [51]. The final scores obtained ranged from 0 to 300.

After a pilot study in 100 PTC cases with available molecular workup, any cytoplasmic positivity with VE1 staining was considered as sufficient to render a case as BRAF-positive on immunostaining. The similar approach was originally described by Bullock et al. [31] and further validated by several independent groups [20,41].

### 4.4. BRAF Sanger Sequencing

Total DNA was extracted from 10 μm thick paraffin-embedded whole tissue sections using a RecoverAll™ Total Nucleic Acid Isolation Kit (Life Technologies, Carlsbad, CA, USA) as per the manufacturer’s instruction. PCR reaction was performed using a primer pair (forward, 5′-TCATAATGCTTGCTCTGATAGGA-3′ and reverse, 5′-GGCCAAAAATTTAATCAGTGGA-3′). Sanger sequencing was performed using the same primers and a BigDye Terminator sequencing kit (Applied Biosystems, Carlsbad, CA, USA) on a 3730xl DNA analyzer (Applied Biosystems), as previously described [54,55].

### 4.5. Digital PCR Analysis 

In discordant cases showing VE1 expression but wild type of *BRAF* using Sanger sequencing, droplet digital PCR was performed using TaqMan dPCR assay (Life Technologies, Carlsbad, CA, USA) and the QuantStudio 3D Digital PCR system (Life Technologies), as described elsewhere. In brief, 6.6 µL of genomic DNA (10–20 ng), 7.5 µL of digital PCR master mix, and 0.9 µL of *BRAF*^V600E^ TaqMan probe was loaded on a digital PCR chip. PCR was performed using the following conditions: one cycle of 96 °C for 10 minutes, 39 cycles of 60 °C for 2 min and 98 °C for 30 s, and one cycle of 60 °C for 2 min. The digital PCR data were analyzed using the Relative Quantification module of the QuantStudio 3D AnalysisSuite Cloud software (Life Technologies). The confidence level was set to 95%, and the desired precision value was 10%.

### 4.6. Statistical Analysis

All the statistical analyses were performed using SPSS 21.0 software (IBM, Armonk, NY, USA). By considering the molecular method as the gold standard for the detection of *BRAF*^V600E^ mutation, we determined the sensitivity, specificity, negative predictive value, and positive predictive value of *BRAF*^V600E^ mutation detection using VE1 IHC. The correlation of clinicopathological features and *BRAF*^V600E^ mutation was analyzed using χ^2^-test or Fisher’s exact test when appropriate. A *p* value of less than 0.05 was considered statistically significant.

## 5. Conclusions

In our series, VE1 IHC was reliable and accurate in the detection of *BRAF*^V600E^ mutation in FFPE PTC specimens. Discordant cases were exceedingly rare; furthermore, all VE1 false positives were resolved using digital PCR, a technique more sensitive than direct sequencing. As such, VE1 IHC could overcome the challenges of Sanger sequencing in FFPE samples.

## Figures and Tables

**Table 1:** Discordant cases.

##	ID	TMA		*BRAF* (Sanger 1st)	First Interpretation	WTS		*BRAF* (Sanger 2nd)	Digital PCR * (Ground Truth)	Conclusion
		VE1 Score	VE1 Result			VE1 Score	VE1 Result			
1	PTC2_33	100	VE1+	wt	VE1-FP	300	VE1+	wt	24.9%, 25.8%	concordant
2	PTC4_22	0	VE1−	V600E	VE1-FN	0	VE1−	V600E	n/a	VE1-FN
3	PTC5_41	0	VE1−	V600E	VE1-FN	0	VE1−	V600E	n/a	VE1-FN
4	PTC7_30	300	VE1+	wt	VE1-FP	300	VE1+	wt	3.1%, 4.2%	concordant
5	PTC8_31	0	VE1−	V600E	VE1-FN	0	VE1−	V600E	n/a	VE1-FN
6	PTC8_39	280	VE1+	wt	VE1-FP	250	VE1+	wt	8.1%; 13.9%	concordant

* digital PCR was performed if repeat BRAF sequencing on matched whole tissue section was negative. FN, false negative; FP, false positive; n/a, not available; PCR, polymerase chain reaction; TMA, tissue microarray; wt, wild type; WTS, whole tissue section.

**Table 2:** Resolving PTC cases discordant between VE1 immunostaining and molecular test.

Source	Year	Molecular Test 1	Discordant, n	Molecular Test 2	VE1-FP after Test 1	VE1-FP after Test 2	VE1-FN after Test 1	VE1-FN after Test 2	Remained Discordant
Bullock [31]	2012	Sseq	11/96 (11%)	re-Sseq, MPSeq	10	3	1	0	3
McKelvie [47]	2013	C-PCR	9/71 (13%)	SNaPshot	9	1	0	0	1
Dvorak [38]	2014	Sseq	7/73 (10%)	re-Sseq, SNaPshot, NGS	4	0 *	3	2	2
Fisher [17]	2014	Pyroseq	4/29 (14%)	re-Pyroseq	4	4	0	0	4
Na [33]	2015	real-time PCR	8/104 (8%)	Sseq, nested PCR	8	4 **	0	0	4
Martinuzzi [19]	2016	Sseq, qPCR	7/86 (8%)	NGS	2	2	5	5 ***	7
Zhu [35]	2016	Sseq	8/118 (7%)	ARMS	8	7	0	0	7
Szymonek [23]	2017	real-time PCR	8/137 (6%)	NGS	6	6	2	2	8
Zhang [44]	2018	real-time PCR	7/132 (5%)	Sseq	7	5	0	0	5
current study	2019	Sseq	6/514 (1.2%)	digital PCR	3	0	3	3	3
TOTAL			75/1360 (5.5%)		61	32	14	12	44/75 (59%)

* two IHC+ cases turned out to be IHC-negative after repeat IHC; ** four IHC+/Seq- cases showed low peaks but were considered discordant by the authors; *** one IHC-/qPCR+ case showed 1% mutant alleles; FN, false negative; FP, false positive; PCR, polymerase chain reaction; Sseq, Sanger sequencing; C-PCR, competitive PCR; NGS, next-generation sequencing; Pyroseq, pyrosequencing; qPCR, quantitative PCR; ARMS, amplification-refractory mutation system; IHC, immunohistochemistry.

## Supplementary Materials

The following are available online at https://www.mdpi.com/2072-6694/12/3/596/s1, Table S1. Correlation between *BRAF*^V600E^ and clinicopathological parameters in 514 patients with papillary thyroid carcinoma, Table S2. Clinicopathological features of three patients with false negative VE1 immunostaining.
