# Supplementary material for: VE1 Immunohistochemistry Improves the Limit of Genotyping for Detecting BRAFV600E Mutation in Papillary Thyroid Cancer
Source: Cancers (Basel). 2020 Mar 5;12(3):596. doi: 10.3390/cancers12030596 (PMC7139976; doi:10.3390/cancers12030596)
Supplement: Supplementary file 1 [file cancers-12-00596-s001.pdf]

Supplementary Materials: VE1 Immunohistochemistry Improves the Limit of Genotyping for Detecting *BRAF*^{V600E} Mutation in Papillary Thyroid Cancer

Sonam Choden, Somboon Keelawat, Chan Kwon Jung and Andrey Bychkov

Table S1. Correlation between *BRAF*^{V600E} and clinicopathological parameters in 514 patients with papillary thyroid carcinoma.

	***BRAF*^{V600E} n = 436 (84.8%)**	***BRAF* Wild Type n = 78 (15.2%)**	***p* Value**
Mean age (y)	47.9 ± 13.2	42.1 ± 14.3	<0.01
Age < 45 y	184 (39.2%)	41 (52.6%)	0.05
Male sex	89 (20.4%)	21 (26.9%)	0.10
Mean tumor size (mm)	16.1 ± 7.7	16.2 ± 6.7	1
Histopathological variant			
Classic	390 (89.4%)	62 (79.5%)	<0.01
Follicular variant, all	6 (1.4%)	11 (14.1%)	<0.01
Encapsulated follicular	0 (0%)	5 (6.4%)	<0.01
Infiltrative follicular	6 (1.4%)	6 (7.7%)	<0.01
Tall cell variant	21 (4.8%)	0 (0%)	0.02
Tall cell variant + tall cell features (>10%)	88 (20.2%)	1 (1.3%)	<0.01
Multifocality	194 (44.5%)	23 (29.5%)	<0.01
Micropapillary component	311 (71.3%)	37 (47.4%)	<0.01
Chronic lymphocytic thyroiditis	139 (31.9%)	35 (44.9%)	0.01
Extrathyroidal extension	336 (77.1%)	41 (52.6%)	<0.01
Gross extrathyroidal extension	92 (21.1%)	6 (7.7%)	0.03
Positive margin	43 (9.9%)	9 (11.5%)	0.33
Vascular invasion	12 (2.8%)	7 (9.0%)	<0.01
Lymphatic invasion	192 (44.0%)	35 (44.9%)	1
Advanced pT stage (pT3 + pT4)	336 (77.1%)	41 (52.6%)	<0.01
Lymph node metastasis	277 (63.5%)	39 (50.0%)	0.01
Lateral neck metastasis (pN1b)	91 (20.9%)	13 (16.7%)	0.20
Distant metastasis	4 (0.9%)	4 (5.1%)	0.01
Advanced AJCC stage, 7th edition (III + IV)	153 (35.1%)	17 (21.8%)	0.01
No evidence of disease (median follow-up 71 months)	365 (83.7%)	62 (79.5%)	0.18

AJCC, American Joint Committee on Cancer.

Table S2. Clinicopathological features of three patients with false negative VE1 immunostaining.

	**Patient 1**	**Patient 2**	**Patient 3**
Age (y)	70	50	27
Sex	Female	Male	Female
Tumor size (cm)	1.2	1.0	1.3
Histopathological variant	Warthin-like	Classic	Classic
Multifocality	Absent	Present	Absent
Chronic lymphocytic thyroiditis	Present	Absent	Absent
Extrathyroidal extension	Absent	Present	Absent
Vascular invasion	Absent	Absent	Absent
Lymphatic invasion	Absent	Present	Present
Pathologic T stage	T1	T3	T1
Lymph node metastasis	Absent	Present	Present
Lateral neck metastasis (pN1b)	Absent	Absent	Absent
Distant metastasis	Absent	Absent	Absent
AJCC stage, 7th edition	1	3	1

AJCC, American Joint Committee on Cancer.

© 2020 by the authors. Licensee MDPI, Basel, Switzerland. This article is an open access article distributed under the terms and conditions of the Creative Commons Attribution (CC BY) license (http://creativecommons.org/licenses/by/4.0/).
